# Supplementary figures and images for: Statin use and breast cancer survival – a Swedish nationwide study
Source: BMC Cancer. 2019 Jan 11;19:54. doi: 10.1186/s12885-018-5263-z (PMC6330431; doi:10.1186/s12885-018-5263-z)

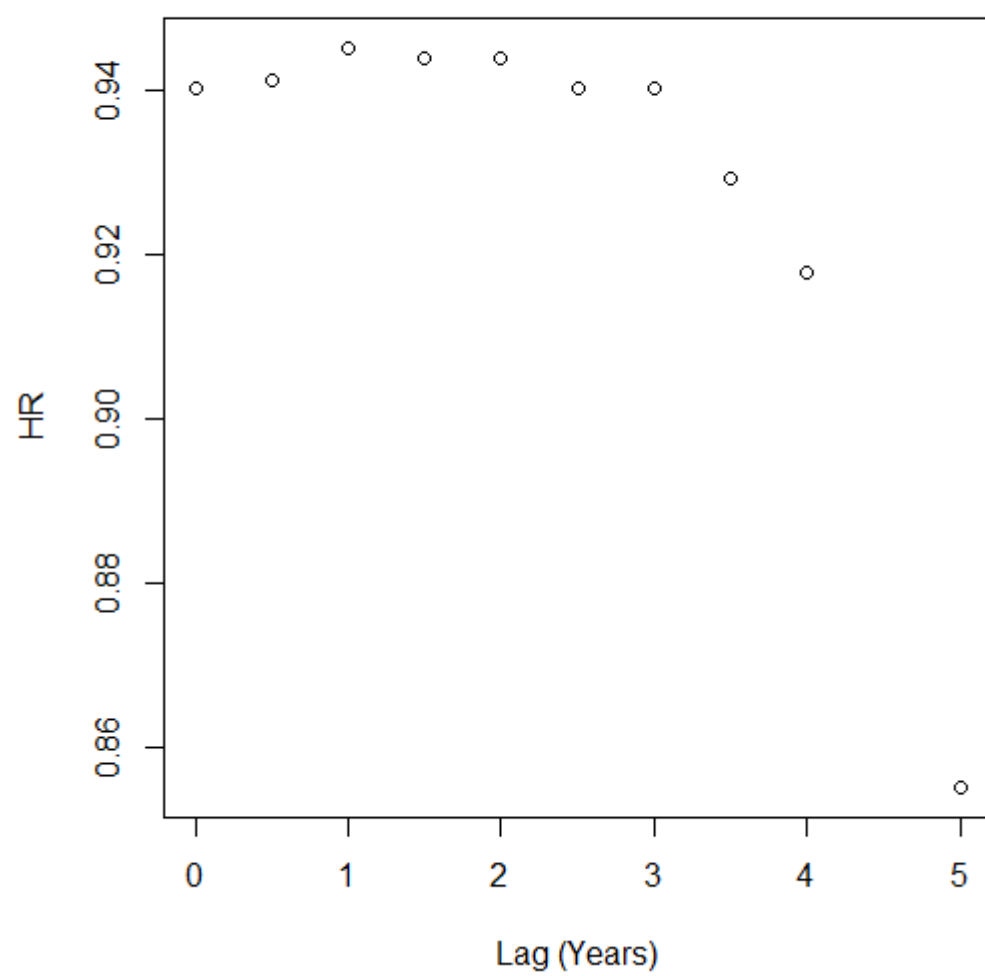

Supplement: Supplementary file 1 — Figure S1. Lagged statin exposure and all-cause mortality. At every lag time marked on the x-axis, the HR has been calculated. One year was decided as lag-time based on calculations lagging exposure, or rather set exposure less than a certain amount equal to zero, not with respect to time, but with respect to cumulative dose. The figure suggests that the statin effect on mortality decreases with increasing lag. (PDF 13 kb) [file 12885_2018_5263_MOESM1_ESM.pdf]

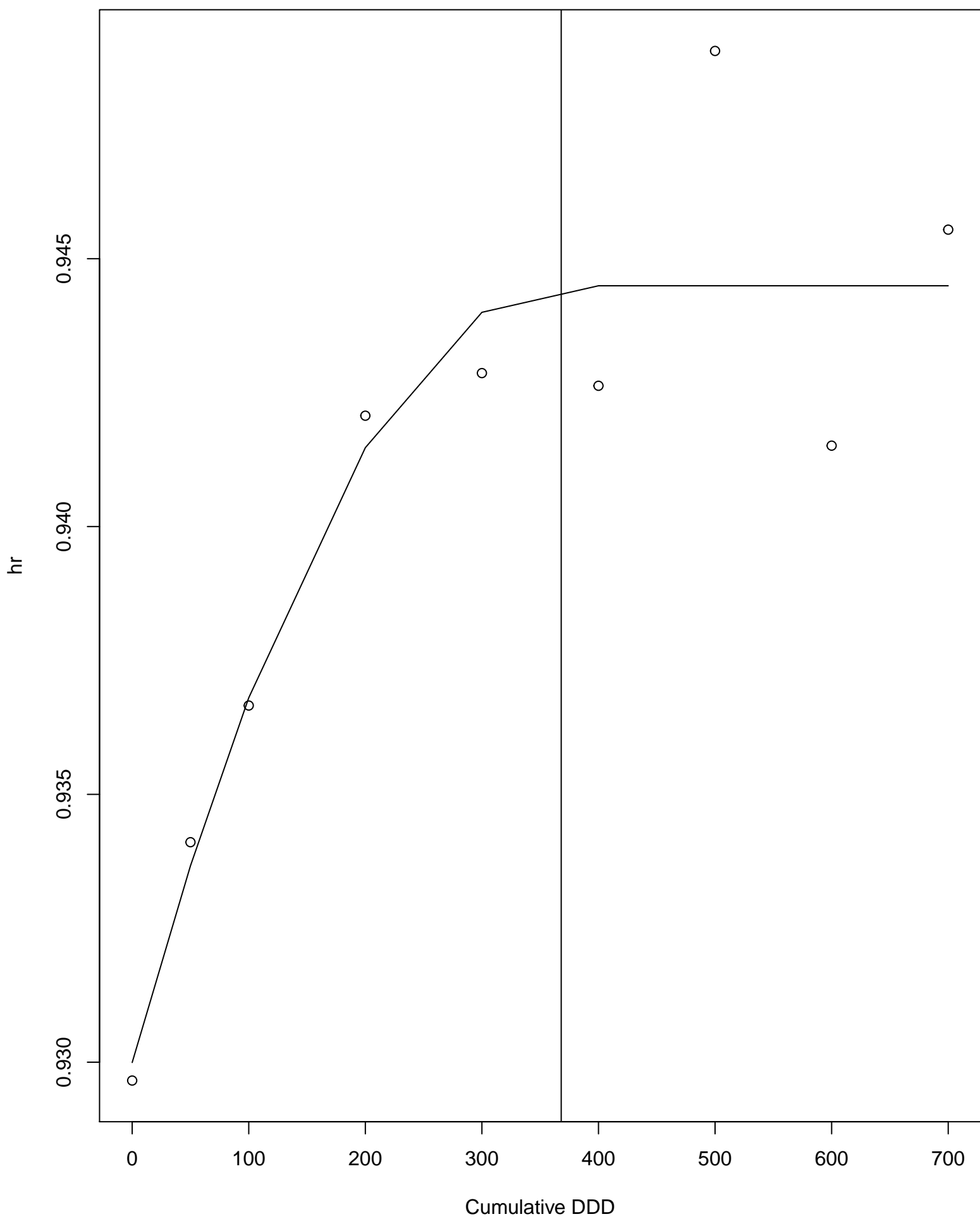

Supplement: Supplementary file 2 — Figure S2. Truncated cumulative statin daily dose and all-cause mortality. Doses below each threshold have been set to zero. The figure indicates that the effect of statin treatment stabilizes after a cumulative dose of roughly 367 DDD. (PDF 8 kb) [file 12885_2018_5263_MOESM2_ESM.pdf]
